# Transcription Factor Analysis in Trypanosomatids

**DOI:** 10.1007/978-1-0716-0294-2_16

**Published:** 2020

**Authors:** Arthur Günzl, Ankita Srivastava, Ujwala Gosavi

**Affiliations:** 1 Department of Genetics and Genome Sciences, UConn Health, Farmington, CT, USA.; 2 Department of Genetics and Genome Sciences, UConn Health, Farmington, CT, USA.

**Keywords:** In vitro transcription, Extract preparation, Primer extension, Radiolabeling of DNA, Promoter pull-down assay

## Abstract

Known transcription factors of trypanosomatid organisms are extremely divergent in amino acid sequence to their counterparts in other eukaryotes. Sequence similarity is so limited that factors have been primarily identified by functional and structural studies. In addition, trypanosomatids may have evolved factors that are specific to this group of organisms. Under these circumstances, an in vitro transcription system is invaluable as it allows for unambiguous determination of a factor’s transcriptional role. Here we describe procedures for the preparation of transcriptionally active extracts, detail in vitro transcription reactions, and specify the particular strategy necessary to detect template-derived RNA in this system. As examples of how to use this system, we describe factor depletion from extract and antibody-mediated interference with a factor’s transcriptional function. Furthermore, we detail a promoter pull-down assay that makes use of the extracts and facilitates analysis of a factor’s interaction with promoter DNA.

## 1 Introduction

Annotation of the completed TriTryp genomes revealed only few transcription factors such as the TATA box binding protein TBP (originally named TRF4), the helicase subunits of transcription factor (TF)IIH, and the TFIIB-related factor 1 [1, 2]. This scarcity of factors correlated with unusual modes of transcription. In trypanosomatids, unrelated protein coding genes are organized in directional gene arrays that are transcribed polycistronically, tRNA genes are associated with small RNA genes driving their expression, and spliced leader (SL) RNA genes are the only noncoding RNA genes transcribed by RNA polymerase (pol) II in monocistronic fashion [3, 4]. However, trypanosomatids have many more transcription factors as originally believed. For example, it now appears that trypanosomatids possess orthologs of each and every general transcription factor that assemble a transcription preinitiation complex at and recruit RNA pol II to the SL RNA gene promoter, comprising the factors SNAPc, TFIIA, TFIIB, TFIIH/E, a TFIIF-like factor, and a mediator complex [5–14]. The amino acid sequences of these factors are extremely divergent from their counterparts in other eukaryotes. This is best illustrated by the trypanosome mediator complex MED-T of which nine subunits were identified [13]. While the factor structurally resembled the head domain of yeast mediator and had the functional properties of a mediator complex, none of the trypanosome subunits exhibited recognizable sequence homology to any of the known mediator subunits of other organisms. Similarly, the class I transcription factor A or CITFA, an initiation factor for RNA pol I-mediated transcription, consists of the dynein light chain LC8 and seven proteins that are conserved only among trypanosomatids [15, 16].

Trypanosomatids likely harbor additional transcription factors which are either too divergent to be identified bioinformatically or are specific to these evolutionary early-branched organisms. For example, the structures of trypanosomatid tRNA and 5S rRNA genes are similar to their counterparts in other eukaryotes, yet TFIIIA and the multisubunit TFIIIC, which are important factors for RNA pol III-mediated transcription of these genes, have not been identified in trypanosomatids so far. Furthermore, the recent identification of promoter elements for the transcription of protein coding gene arrays in *Trypanosoma brucei* [17] and cell cycle-regulated, gene-specific promoters in *Leishmania donovani* [18] suggest the presence of yet to be identified DNA-binding proteins that facilitate transcription from these promoters. Finally, *T. brucei* harbors a multifunctional RNA pol I that transcribes the large rRNA gene unit (*RRNA*) as in other organisms as well as the genes of its major cell surface proteins VSG and procyclin [19]. The *RRNA*, VSG, and procylin gene promoters differ structurally, and the latter two are developmentally regulated, indicating that additional factors to CITFA are required to productively initiate transcription from these promoters [20].

There are several approaches to analyze a putative transcription factor in vivo. Chromatin immunoprecipitation (ChIP) assays can reveal whether a protein occupies promoters, and gene silencing or conditional gene knockout experiments combined with RNA analysis may uncover effects of factor depletion on RNA abundances. For transcription factor analysis, it is most telling when nascent or newly synthesized RNA is investigated. In this respect, it should be noted that metabolic labeling of RNA with 4-thiouridine has been successfully applied in *T. brucei* [21]. In addition, a permeabilized cell system has been established in *T. brucei* in which radiolabeled ribonucleotides or bromo-UTP are efficiently incorporated into newly synthesized RNA [22, 23]. However, caveats of these methods are that they either do not provide functional transcription data (ChIP) or cannot rule out that observed effects on RNA abundance are secondary in nature. This is particularly problematic when the factor under investigation does not exhibit any sequence similarity to known factors from model organisms and does not harbor a recognizable DNA-binding domain. An in vitro transcription system, however, provides the means to explicitly determine the transcriptional function of a factor. The key step in establishing such a system is the generation of transcriptionally active, cell-free extracts. This has been pioneered in *T. brucei* with a nuclear extract that supported accurately initiated transcription by RNA pol III [24] and of SL RNA genes by RNA pol II [25]. A slightly modified extract established a comparable system in another trypanosomatid, *Leptomonas seymouri* [26]. Furthermore, a *T. brucei* “whole-cell extract” (WCE), in which nuclear proteins were extracted into the soluble cytoplasmic fraction, proved to be active in SL RNA gene and RNA pol I-mediated transcription [27, 28]. While these extracts were established on a large scale in procyclic trypanosomes, the WCE could also be achieved on a small scale and was successfully applied to bloodstream form trypanosomes [29] and therefore may be the method of choice for trypanosomatid species that cannot be easily grown to large numbers. A transcription system for protein coding gene arrays, however, has not been established yet.

**Fig. 1:**
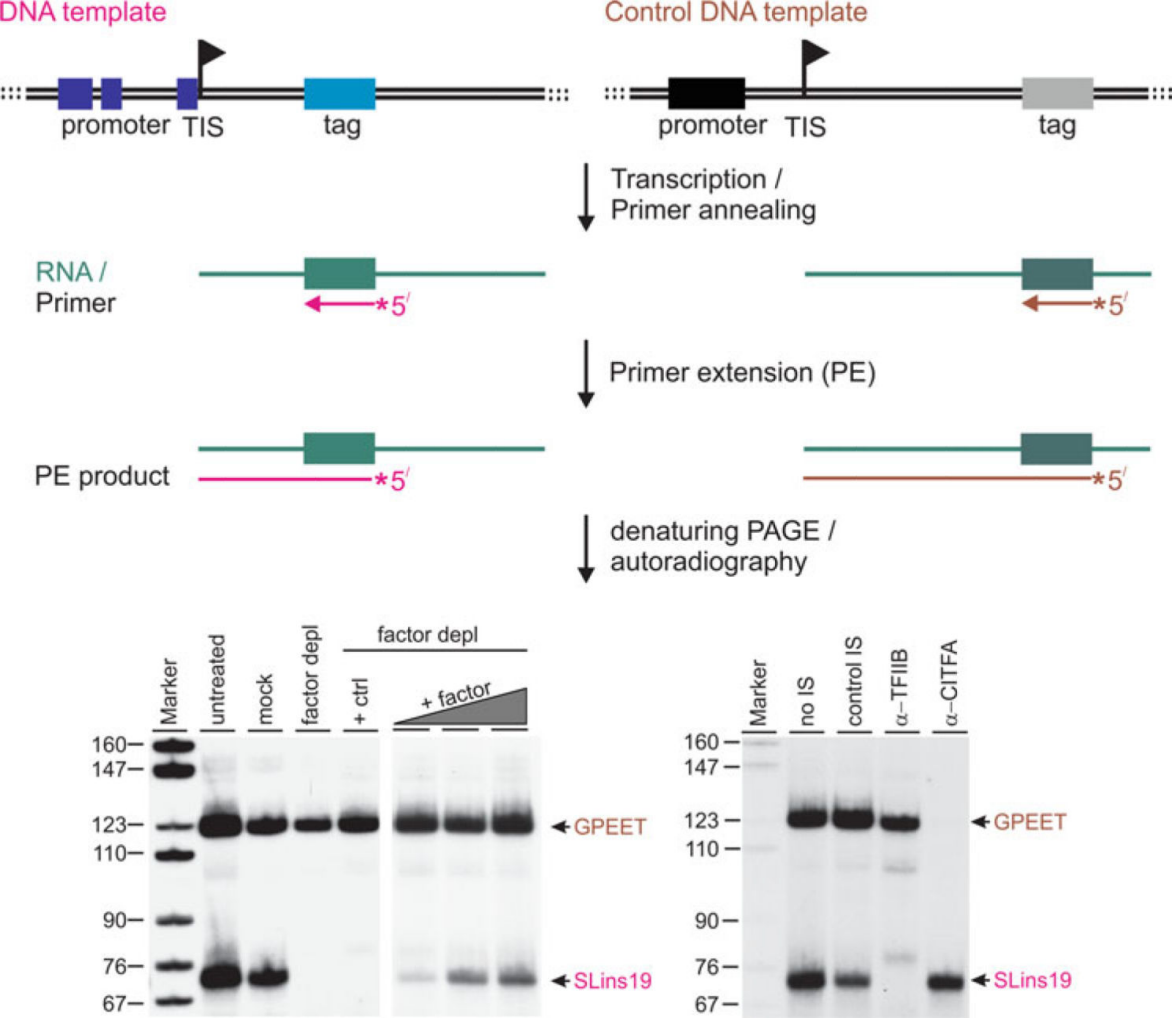
In vitro transcription assay. Template DNAs harbor a promoter region with essential sequence elements (dark blue and black rectangles), the transcription initiation site (TIS, indicated by a flag), and unrelated tag sequences downstream of the TIS. The tags are placed at different distances from the flag to generate differently sized transcription signals. After the transcription reaction, total RNA is prepared and radiolabeled, tag-complementary oligonucleotides are annealed to template-derived transcripts. Reverse transcription extends these primers until the enzyme reaches the 5′ ends of the RNAs. Finally, radiolabeled extension products are separated on denaturing 50% urea/6% PAA gels and visualized by autoradiography. The bottom panels show cotranscription of the SLins19 and GPEET-trm templates. SLins19 contains the RNA pol II-recruiting promoter of the SL RNA gene, and GPEET-trm the RNA pol I-specific GPEET procyclin promoter template. On the left, an essential basal factor for RNA pol II transcription was depleted from extract (factor depl) which specifically abolished SLins19 transcription. Mock treatment of extract with control beads did not have this specific effect. Adding back purified factor (+ factor) partially restored SLins19 transcription in a dose-dependent manner whereas a similarly derived control factor (+ ctrl) did not restore transcription. As shown on the right, treatment of extract with anti-TFIIB immune serum (α-TFIIB) or with anti-CITFA serum (α-CITFA) abolished SLins19 and GPEET-trm transcription, respectively, whereas a nonspecific immune serum (control IS) had no effect on GPEET transcription and marginally affected SLins19 transcription

A key problem in preparing trypanosome extracts is that the cells are difficult to break due to their robust microtubule-based cytoskeleton and their spindle-like cell shape. The harsh treatments necessary to achieve quantitative cell breakage lead to genomic DNA contamination of extracts that is difficult to eliminate. While the use of a labeled ribonucleotide and a specific DNA template generates a detectable RNA of defined size in cell-free systems of other organisms, the addition of a labeled ribonucleotide to trypanosome extracts results in RNA smears even in the absence of template DNA [24]. The strategy to circumvent this general labeling activity and specifically detect template-derived RNA is based on the insertion of a ~20 bp-long, unrelated oligonucleotide tag sequence ~50–120 bp downstream of the transcription initiation site (TIS) in template DNA. The resulting transcripts can then be specifically detected by primer extension assays (Fig. 1). Moreover, since the reverse transcriptase extends a primer to the 5′ end of an RNA, the size of the extension product reveals whether transcription initiation on the template was accurate. For *T. brucei*, tagged templates were generated and successfully transcribed for the SL RNA gene (SLins19) and the RNA pol I-transcribed rRNA (Rib-trm), VSG (VSG-trm), metacyclic (m)VSG (1.61-trm, 1.63 trm), and procyclin genes (GPEET-trm, originally termed PARPtrm) [25, 27, 30].

Once the in vitro system for a particular promoter/gene is established, it offers various ways to probe or confirm the function of a putative transcription factor. For example, the conditional gene silencing system in *T. brucei* [31] enables the comparison of transcriptional activities in extracts from uninduced cells and from cells in which a factor has been depleted [12, 32]. If gene silencing is not an option, removal of a factor from extract by immunoprecipitation is an alternative and can abolish transcription of a specific template in vitro (Fig. 1, bottom left) [9, 12–14, 32]. Finally, preincubation of extract with an antibody against the endogenous factor often interferes with its function, affecting transcription (Fig. 1, bottom right) [9, 12, 14, 32].

**Fig. 2:**
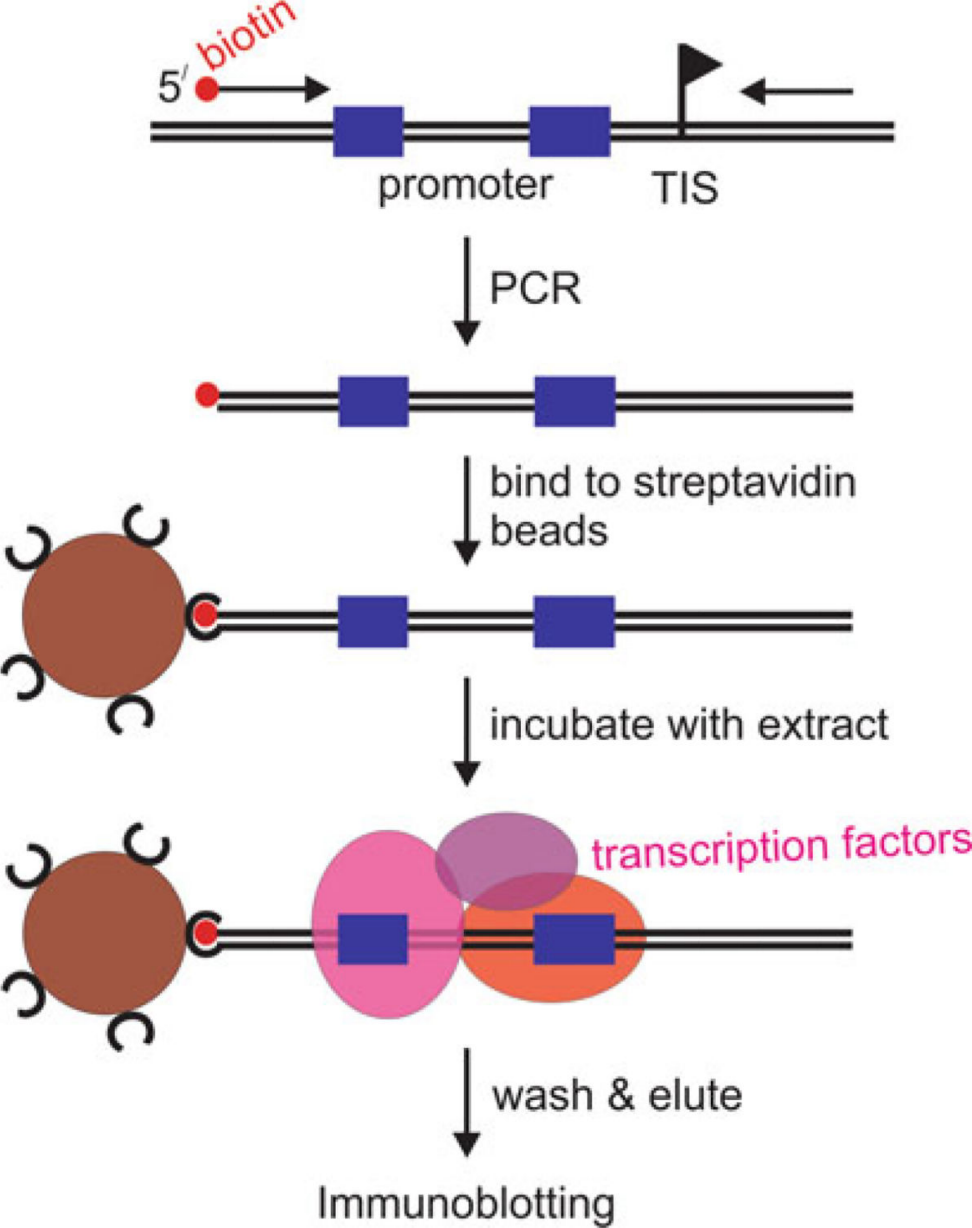
Schematic outline of the promoter pull-down assay. A promoter region is amplified by PCR with one oligonucleotide carrying a biotin group at its 5′ end. The biotinylated promoter DNA is coupled to streptavidin beads and incubated with extract under transcription conditions. After washing the beads, DNA-bound proteins are eluted in SDS buffer and analyzed by standard immunoblotting. Blue rectangles and the flag denote promoter sequence elements and the transcription initiation site (TIS), respectively

Another use of extracts is the promoter pull-down assay, which can reveal the interaction between a transcription factor and its cognate promoter (Fig. 2). The assay does not require cross-linking of proteins and is less stringent than an electric mobility gel shift assay (EMSA). For example, EMSA could not be established with factors that bind to the SL RNA gene promoter whereas the promoter pull-down assay readily revealed binding of a SNAPc subunit to this promoter [33]. While meaningful EMSAs often require a purified fraction of the transcription factor, promoter pull-downs typically work well with crude extracts. However, the latter assay requires detection of the factor by immunoblotting. Hence, it is only feasible if an antibody against the factor is available or extract is prepared from cells that express a tagged version of the factor that can be detected through the tag. Once established, the pull-down assay can be used to probe the DNA–protein interaction of a factor by mutating promoter elements.

## 2 Materials

All buffers should be prepared with RNase-free water and analytical grade reagents.

### 2.1 Extract Preparation

1. 5 L round, flat-bottomed glass flask, stirrer, and stirring magnet.
2. 15 ml “tight” glass Dounce homogenizer (Wheaton, Millville, NJ, USA) for large-scale preparations (*see* Note 1).
3. 800 μm Low Binding Silica Beads (OPS Diagnostics, Lebanon, NJ, USA) for small-scale preparations.
4. 15- and 50-ml Falcon tubes.
5. Amicon Ultra-4 centrifugal filtering unit (Millipore Sigma,St. Louis, MO, USA) with a molecular weight cutoff of 10 kDa.
6. A Dewar with liquid nitrogen for shock-freezing of extract aliquots (alternatively, a dry-ice bath can be used).
7. Tryp wash: 100 mM NaCl, 3 mM MgCl_2_, 20 mM Tris–HCl pH 7.5.
8. Transcription buffer (incomplete): 150 mM sucrose, 20 mM L-glutamic acid, 10 mM HEPES–KOH pH 7.7, 3 mM MgCl_2_. Complete the buffer just before use by making it 1 mM dithiothreitol (DTT), 10 μg/ml leupeptin, and 10 μg/ml aprotinin (complete transcription buffer aliquots can be stored at −20 °C and should be kept on ice after thawing) (*see* Note 2).
9. EDTA-free complete protease inhibitor cocktail (Millipore Sigma): Dissolve one tablet in 1 ml incomplete transcription buffer, aliquot, and store at −20 °C.
10. HS (high-salt) buffer (store aliquots at −20 °C): 150 mM sucrose, 1.5 M KCl, 20 mM Hepes–KOH pH 7.7, 3 mM MgCl_2_.
11. 1 M dithiothreitol (DTT) dissolved in water and stored at −20 °C in 1 ml aliquots.
12. 3 M KCl.

### 2.2 In Vitro Transcription Analysis

#### 2.2.1 In Vitro Transcription Reaction and RNA Preparation

1. Template DNA (1 μg/μl) in the form of a plasmid (*see* Note 3) and a standard nontemplate plasmid DNA such as pUC19 (1 μg/μl).
2. Transcription buffer (*see* above).
3. 10× salts: 0.2 M L-glutamic acid, 0.2 M Hepes–KOH pH 7.7, 30 mM MgCl_2_, 72 mM DTT, 3.5 mM EDTA, 8.9 mM EGTA, 100 μg/ml leupeptin, 100 μg/ml aprotinin. Prepare a 1-ml aliquot and store at −20 °C.
4. 25% polyethylene glycol (PEG; w/v). Prepare 1-ml aliquots and store at −20 °C.
5. 0.5 M creatine phosphate. Store in 0.1 ml aliquots at −20 °C.
6. 12 mg/ml creatine kinase. Prepare freshly every time with incomplete Transcription buffer. Creatine kinase activity is highly unstable and will not survive a freeze–thaw cycle.
7. 10 mM NTP mix (10 mM of ATP, CTP, GTP, UTP; 100 mM stocks of each nucleotide are available from Millipore Sigma [Roche]).
8. TRIzol™ LS Reagent (ThermoFisher Scientific, Grand Island, NY, USA).
9. 20 mg/ml Glycogen, RNase-free (Millipore Sigma [Roche]).
10. DNase I (10 U/μl), RNase-free (Millipore Sigma [Roche]); includes 10× DNase I buffer.
11. Buffered phenol–chloroform–isoamyl alcohol (25/24/1, v/v/v) and chloroform.
12. 100% ethanol, 70% ethanol, 3 M sodium acetate (NaOAc) pH 7.0.

#### 2.2.2 Factor Depletion from Extract

1. IgG Sepharose® 6 Fast Flow (Millipore Sigma).
2. Transcription buffer (*see* above).

#### 2.2.3 Antibody-Mediated Transcription Inhibition

1. Immune serum or purified antibody against the endogenous transcription factor to be analyzed.
2. Preimmune serum and/or nonspecific immune serum/antibody of same origin.

#### 2.2.4 Radiolabeling of Oligonucleotides and DNA Marker

1. Oligonucleotide to be labeled (100 ng/μl).
2. T4 Polynucleotide Kinase (PNK) (New England Biolabs, Ipswich, MA, USA); includes 10× PNK buffer.
3. [γ-^32^P]ATP, 6000 Ci/mmol (PerkinElmer, Waltham, MA, USA).
4. Marker pBR322, *Msp*I-digested (1 μg/μl, New England Biolabs).
5. DNA Polymerase I, Large (Klenow) Fragment (New England Biolabs), includes 10× Klenow buffer.
6. [α−^32^P]dCTP, 3000 Ci/mmol (PerkinElmer).
7. 5 mM dGAT mix (5 mM of dGTP, dATP, dTTP) (100 mM stocks of each nucleotide are available from Millipore Sigma [Roche].
8. Micro Bio-Spin 6 columns (Bio-Rad, Hercules, CA, USA).
9. Čerenkov counter.

#### 2.2.5 Primer Extension Reaction and Denaturing Polyacrylamide Gel Electrophoresis (PAGE)

1. SuperScript Reverse Transcriptase (ThermoFisher Scientific), includes 5× first strand buffer and 0.1 M DTT aliquots.
2. 10 mM dNTP mix.
3. 5′−^32^P-endlabeled oligonucleotide(s) (*see* Subheading 3.2.4).
4. Radiolabeled DNA marker (*see* Subheading 3.2.4).
5. 10×/1× Tris–Borate–EDTA (TBE) buffer: For 1 L of 10× TBE buffer dissolve 108 g of Tris base and 55 g of boric acid in 900 ml of water. Add 40 ml of 0.5 M EDTA, pH 8.0. Make up volume to 1 L, autoclave and store at room temperature.
6. Urea loading buffer: 50% (w/v) urea in 1× TBE buffer, 0.1% (w/v) xylene cyanol, 0.1% (w/v) bromophenol blue.
7. System for vertical gel electrophoresis which includes glass plates (~18 cm × 20 cm), 0.4 mm thick spacers and comb, electrophoresis chamber, and a high voltage power supply.
8. 50% urea/1× TBE solution: dissolve 500 g urea in 100 ml 10× TBE buffer and water to a volume of 1 L, filter solution and store at room temperature in a dark glass bottle.
9. 50% urea/20% PAA/1× TBE solution: dissolve 500 g urea in 500 ml of 40% polyacrylamide (PAA) solution (29:1, Fisher Scientific, Fair Lawn, NJ, USA), 100 ml 10× TBE buffer and water to a volume of 1 L, filter solution and store at room temperature in a dark glass bottle.
10. 10% ammonium persulfate (APS, w/v), prepare a 1-ml aliquot and store at 4 °C up to 1 week.
11. Tetramethylethylenediamine (TEMED).
12. Heating block.
13. Whatman paper.
14. Vacuum gel dryer.
15. X-ray film (Research Products International, Mt. Prospect, IL, USA), intensifying screens, film cassettes and film developer.

### 2.3 Promoter Pull Down Assay

1. Oligonucleotides for promoter DNA amplification, one of which should be synthesized with a 5′–terminal biotin group.
2. QIAquick Gel Extraction Kit (Qiagen, Germantown, MD,USA).
3. Paramagnetic Streptavidin Dynabeads M-280 (ThermoFisher).
4. 2x B&W buffer: 10 mM Tris–HCl, pH 7.5, 1 mM EDTA, 2 M NaCl.
5. A magnetic stand to collect paramagnetic beads, for example the Magna GrIP™ Rack (Millipore Sigma).
6. TK_20_ buffer: 150 mM sucrose, 20 mM Hepes–KOH pH 7.7, 20 mM potassium L-glutamate, 20 mM KCl, 3 mM MgCl_2_, 2.5% (w/v) polyethylene glycol, 0.2 mM EDTA, 0.5 mM EGTA, 4 mM DTT, 10 μg/ml leupeptin, 10 μg/ml aprotinin.
7. Bovine serum albumin.
8. Polyvinylpyrrolidone (PVP10, Millipore Sigma).
9. TN_40_ buffer: 150 mM sucrose, 20 mM Tris–HCl pH 8.0, 40 mM NaCl, 3 mM MgCl_2_, 0.5 mM DTT, 10 μg/ml leupeptin, 10 μg/ml aprotinin.

## 3 Methods

### 3.1 Extract Preparation

Several techniques have been used to break trypanosome cells including Stansted Cell Disruptor and French Press. However, only two procedures reliably produced transcriptionally active extracts, namely repeated cycles of vortexing cells in the presence of glass beads followed by shock-freezing and thawing the sample (small scale), and the use of a glass Dounce homogenizer (large scale). For extract preparations, all solutions should be ice-cold and Eppendorf and Falcon tubes precooled on ice before use.

#### 3.1.1 Small-Scale WCE Preparation

1. Before harvesting cells, place a vortexer and the liquid nitrogen Dewar in a cold room. Wash an Amicon Ultra-4 centrifugal filtering unit twice by passing 2 ml of water through the filter device at 4000 × *g*. This washing step is important because the filter unit contains trace amounts of glycerol that needs to be removed.
2. Grow 2–3 × 10^9^ trypanosomes to mid-log phase and pellet cells at 2700 × *g* and 2 °C for 10 min. Discard the supernatant. Resuspend cells in 10 ml of Tryp wash, transfer cells to a 15-ml Falcon tube, and spin as before. Repeat the washing step once with Tryp wash and once with incomplete Transcription buffer, increasing the centrifugation speed to 3300 × *g* in the last step to counter the viscosity of the sucrose-containing Transcription buffer. Record packed cell volume (PCV; should be around 0.25 ml).
3. Resuspend cells in one PCV of complete Transcription buffer, transfer cells to a precooled 1.5-ml microcentrifuge tube, and incubate cells on ice for 20 min. The Transcription buffer is slightly hypotonic, causing the cells to swell moderately. During incubation, equilibrate the glass beads in incomplete Transcription buffer, cut the top of a blue 1-ml tip and transfer 0.2 ml of beads to the cell suspension. Just prior to breaking the cells, mix 20 μl of protease inhibitor cocktail into the suspension.
4. Cells are broken in the cold room by five cycles of vortexing, shock-freezing and thawing the suspension. Vortex the cell suspension at full power for 1 min, let it sit on ice for 1 min and then repeat the vortexing step. Shock-freeze the suspension in liquid nitrogen and thaw it in your hands for the next cycle.
5. For the protein extraction procedure, have a precooled 1.5-ml tube containing 60 μl of ice-cold HS buffer ready. Take off the broken cell suspension from the glass beads all at once (best by pushing a yellow tip on top of a blue tip) and rapidly mix the suspension with the HS buffer aliquot. The rapid mixing with high salt buffer will minimize nuclear lysis. Incubate the broken cell suspension for 20 min on ice and intermittently (~three times) flick/invert the tube during this period.
6. To separate the extract from nuclei and insoluble cell debris,spin the tube in a cooling microcentrifuge at 25,000 × *g* and 2 °C for 10 min. Transfer the extract (~500 μl) to a new precooled tube using a 200 μl-pipette.
7. Reduce the salt concentration of the extract by adding an equal volume of complete Transcription buffer, and concentrate the extract in the prewashed Amicon Ultra-4 filter unit at 4000 × *g* and 2 °C to a volume of 150–200 μl.
8. Prepare 50-μl aliquots in precooled 1.5 ml-tubes, shock-freeze the extract, and store at −80 °C.

#### 3.1.2 Large-Scale WCE Preparation

1. Grow 4 L of procyclic trypanosomes (*see* Note 4) to mid-log phase (1 × 10^7^ cells/ml) in a 5 L round flat-bottom glass flask using a stirrer and a stirring magnet. Harvest cells in 500-ml centrifugation bottles at 3000 × *g* and 2 °C for 10 min. Resuspend cell pellets in 5 ml of Tryps wash each. Transfer and combine the cell suspensions in a 50-ml Falcon tube. Pellet cells again at 3000 × *g* and 2 °C for 7 min and discard the supernatant without disturbing the cell pellet.
2. Wash cells twice with 40 ml of Tryps wash and spin as above. Determine the packed cell volume (PCV), which should be 5–7 ml.
3. Resuspend and wash cells once in 30 ml of ice-cold incomplete transcription buffer. Centrifuge at 3300 × *g* and 2 °C for 10 min. The increase in centrifugation speed is necessary to counter the viscosity of the sucrose-containing transcription buffer. Remove the supernatant and resuspend cells in one PCV of incomplete transcription buffer so that the total volume is ~10–14 ml. Incubate the cells on ice for 20 min.
4. During this incubation, prepare the glass Dounce homogenizer by washing it once with 100% ethanol, three times with water, and once with incomplete Transcription buffer. Place the homogenizer in an ice bucket so that it touches the bottom. In this way, it cannot break through the ice during douncing. Transfer cells to the homogenizer.
5. Prior to douncing, add DTT to a final concentration of 1 mM and leupeptin and aprotinin to final concentrations of 10 μg/ml. Dilute a 2 μl sample of the cell suspension in 38 μl of Tryps wash for microscopic analysis.
6. Vigorously dounce the solution until about 70–80% of the trypanosomes are broken. Dependent on the tightness of the homogenizer, this may take 3–10 min. To assess cell breakage, take 2 μl samples, dilute and compare them to the unbroken cell sample under the microscope at 400× magnification.
7. Prepare 1-ml aliquots of broken cell suspension, shock-freeze them in liquid nitrogen and store them at −80 °C. Shock-freezing is an important step since it appears to disrupt cells that have not been broken by douncing.
8. Transcription extract can be prepared from one aliquot at a time. Thaw a 1-ml aliquot of broken cell suspension in your hands and place on ice. Add 100 μl of HS buffer to a new tube, then transfer and rapidly mix the broken cell suspension with HS buffer to minimize nuclear lysis. Incubate the extract on ice for 20 min and flick/invert the tubes during this period three times.
9. Separation of extract from nuclei and cell debris, dilution and concentration of extract, and preparation and storage of transcription extract aliquots should be carried out as detailed in Subheading 3.1.1, **steps 6**–**8**. Note that the soluble extract before dilution is ~600 μl in this procedure and the final volume of the extract after concentration should be between 200 and 250 μl.

#### 3.1.3 Nuclear Extract Preparation (Large Scale)

The preparation of 1-ml aliquots of broken cell suspension is identical to the previous protocol (Subheading 3.1.2, **steps 1**–**7**). For nuclear extract preparation a single 1-ml aliquot of broken cell suspension is treated as follows:

8. Spin the suspension at 16,000 × *g* and 2 °C for 10 min. Discard the supernatant and wash pellet with 900 μl of complete Transcription buffer by gently resuspending the pellet, inverting and flicking the tube. Do not use the pipette for this purpose because nuclei may get damaged.
9. Repeat the spin, discard the supernatant, and resuspend the pellet in 520 μl of complete transcription buffer. For extraction, mix in, drop-by-drop, 80 μl of 3 M KCl (final KCl concentration of 400 mM). Incubate the extract on ice for 20 min and flick/invert the tubes during this period three times.
10. Except for the fact that this extract needs to be diluted with 2.5 volumes of complete Transcription buffer instead of an equal volume, separation of extract from nuclei and cell debris, dilution and concentration of extract, and preparation and storage of extract aliquots should be carried out as detailed in Subheading 3.1.1, **steps 6**–**8**. Note that the nuclear extract has a lower protein concentration and should be more concentrated than WCE (e.g., from ~600 μl before dilution to a final volume of 150 to maximal 200 μl).

### 3.2 In Vitro Transcription Analysis

The method consists of the in vitro transcription reaction, the subsequent RNA analysis by primer extension, and separation and detection of the radiolabeled extension products by denaturing PAGE and autoradiography, respectively. Accordingly, we provide protocols for radiolabeling of oligonucleotides and the DNA size marker pBR322-*Msp*I. In addition, we describe how to deplete an extract of a factor and use immune sera to inactivate a factor in extract.

#### 3.2.1 In Vitro Transcription Reaction and RNA Preparation

1. Thaw or prepare all reagents and place on ice. Set a water bath at 28 °C.
2. Set up the following 38-μl reaction (*see* Note 5): 1.6 μl of template plasmid DNA (do not use more than 1 μg of a single template in a reaction; if there is only one template per reaction, then add nontemplate DNA to a final total DNA amount of 1.6 μg [*see* Note 6]), 2.24 μl of 10× salts, 4 μl of PEG, 1.6 μl of creatine phosphate, 1.6 μl of creatine kinase, 10.96 μl of water, 8 μl of complete Transcription buffer, and 8 μl of extract. Incubate the reaction for 10 min on ice.
3. Add 2 μl of NTP mix to the reaction and incubate transcription reaction for 1 h at 28 °C.
4. Stop the reaction and prepare total RNA from the reaction as follows: add 60 μl of water, 300 μl of TRIzol reagent, and 60 μl of chloroform to the reaction, vortex the sample for 1 min and incubate on ice for 10 min. Separate the lower red organic phase from the upper colorless aqueous phase by centrifugation in a microcentrifuge at top speed and room temperature for 5 min. Transfer the aqueous phase (200 μl) to a new microcentrifuge tube and mix well with 1 μl of glycogen (glycogen serves as a carrier for efficient RNA precipitation) and 600 μl of ethanol. Precipitate RNA by spinning the sample in a microcentrifuge at top speed and room temperature for 10 min. Discard the supernatant, briefly spin again and remove trace amounts of liquid. Wash the pellet twice with 70% ethanol and air-dry pellet for 5–10 min.
5. To remove all template DNA from the RNA preparation, it is important to treat the sample with DNase as follows: Dissolve RNA pellet in 90 μl of water at 65 °C for 10 min, vortex sample, and place the tube back on ice. Mix the sample with 10 μl of 10× DNase I buffer and 1 μl of DNase I. Incubate sample at 37 °C for 1 h. Stop the reaction and extract RNA with 100 μl of buffered phenol–chloroform–isoamyl alcohol and, subsequently, with 100 μl of chloroform. Transfer the aqueous phase to a new tube, precipitate RNA with 30 μl of 3 M NaOAc and 600 μl of 100% ethanol, and wash RNA pellet twice with 70% ethanol. Air-dry the pellet and proceed to the primer extension reaction. Please note, that RNA preparations are best stored in 70% ethanol (second wash) at −20 °C.

#### 3.2.2 Factor Depletion from Extract

A factor can be depleted from extract at different preparation steps by standard immunoprecipitation (IP). We have obtained best results when extract was subjected to IP after extraction and before dilution with transcription buffer to reduce the salt concentration (between **steps 6** and **7** of Subheading 3.1.1). Furthermore, for factor depletion we routinely prepared extracts from cell lines that exclusively expressed a transcription factor fused to a PTP tag. The PTP tag is a composite tag for tandem affinity purification and includes tandem protein A domains [34]. Hence, IgG beads can be used for efficient removal of a PTP-tagged factor from extract. However, any bead-bound antibody that efficiently precipitates a factor can be used instead. To ensure that the IP procedure does not inactivate the transcription reaction nonspecifically, mock depletion with control beads (e.g., protein A or protein G beads) and cotranscription of an unaffected template are valuable controls (Fig. 1). The gold standard though is when adding back the factor to extract—either in recombinant form or from an independent purification (*see* Note 7)—activates transcription in a dose-dependent manner (Fig. 1).

1. Wash and equilibrate 50 μl of settled IgG beads (100 μl of 1:1 slurry) twice with 0.8 ml complete Transcription buffer, pelleting the beads at 3000 × *g* and 4 °C for 1 min.
2. Add undiluted extract (~500 μl) to beads and slowly rotate tube at 4 °C for 1 h. Spin sample at 3000 × *g* and 2 °C for 2 min. Transfer supernatant, that is, depleted extract, to a new, precooled tube and continue with extract preparation by diluting the extract with complete Transcription buffer as described in Subheading 3.1.1, **step 7**.

#### 3.2.3 Antibody-Mediated Transcription Inhibition

1. The 40-μl in vitro transcription reaction is carried out as described in Subheading 3.2.1 with the following modification: 6 μl of extract (instead of 8 μl) is mixed with 1 μl of a polyclonal immune serum (*see* Note 8) directed against the factor and 1 μl of complete Transcription buffer, and incubated on ice for 30 min. The extract is then added to the transcription reaction and the procedure carried out as described. As an essential control, a reaction with preimmune or a nonspecific immune serum of the same origin should be carried out in parallel.

#### 3.2.4 Radiolabeling of Oligonucleotides and DNA Marker

Please note that working with radioactive nucleotides standardly requires special training of personnel and institutional approval of a radiation safety protocol, specifying where and how experiments are conducted, radioactivity is monitored and radioactive waste is disposed (*see* Note 9 for nonradioactive alternatives).

1. For ^32^P-5′-end labeling of an oligonucleotide to be used in primer extension reactions, set up the following 20 μl reaction: 14.3 μl of water, 2 μl of 10× PNK buffer, 2 μl of oligonucleotide solution (100 ng/μl), 1.2 μl of [γ-^32^P]ATP, and 0.5 μl of PNK. Incubate at 37 °C for exactly 30 min.
2. Separate unincorporated radionucleotides from oligonucleotides by gel filtration using Micro Bio-Spin 6 columns as follows: Remove tip and cap of the column, place it in a 2 ml microcentrifuge tube (tubes come with the columns), and dry column with a 2 min spin at 1100 × *g* and room temperature. Wash the column with 600 μl of water, repeating the spin. Transfer the PNK reaction to the center of the dried column, place the column in a fresh 1.5 ml microcentrifuge tube and spin at 1100 × *g* and room temperature for 4 min. Discard the column and dilute the oligonucleotide solution with 150 μl of water. Store at −20 °C.
3. Radiolabeling of a DNA size marker will allow for direct comparison of primer extension products with DNA fragments of known size in denaturing PAGE. *Msp*I-digested pBR322 can be labeled with radiolabeled dCTP due to 5′-CG overhangs that can be filled in with Klenow enzyme. Set up a 20-μl labeling reaction as follows: 1.0 μl of digested DNA marker, 1 μl of 5 mM dGAT mix, 2 μl of 10× Klenow buffer, 10 μl of water, 5 μl of [α−^32^P]dCTP, and 1 μl of Klenow enzyme. Incubate reaction at 37 °C for 30 min and remove unincorporated nucleotides via a Micro Bio-Spin 6 column as described in **step 2**. Store at 20 °C.

#### 3.2.5 Primer Extension Reaction and Denaturing PAGE

1. Resuspend the dried RNA pellet generated from the in vitro transcription reaction (Subheading 3.2.1) in 11.5 μl of water, and add 4 μl of 5× first strand buffer and 1 μl of labeled oligonucleotide (10.5 μl water/2 μl oligonucleotide when two primers are used in parallel). Anneal primers to RNA by heating the sample to 70 °C for 5 min, followed by an incubation on ice for 5 min.
2. Set up reverse transcription reaction by adding 2 μl of 0.1 M DTT, 1 μl of 10 mM dNTP mix, and 0.5 μl of reverse transcriptase to the annealing reaction. Incubate at 42 °C for 45 min.
3. Precipitate labeled DNA by mixing the reaction with 2.5 μl of 3 M NaOAc and 60 μl of 100% ethanol. Spin sample for 7 min at top speed and room temperature in a microcentrifuge, remove all liquid, air-dry the pellet and resuspend DNA in 10 μl of Urea loading buffer.
4. Dilute labeled DNA marker (Subheading 3.2.4, **step 3**) in Urea loading buffer such that a 5 μl sample generates about 20 counts per second in a Čerenkov counter.
5. For denaturing PAGE, assemble two glass plates with spacers and seal the plates with packaging tape except for the top part. For a 6% PAA gel (*see* Note 10), mix 7.5 ml of 50% urea/20% PAA/1×TBE solution with 17.5 ml of 50% urea/1× TBE solution, 0.15 ml of 10% APS, and 15 μl of TEMED. Immediately pour the gel, insert the comb and let the gel polymerize for at least 30 min in horizontal position. Clamps may be used to tighten the plates during polymerization.
6. After removing tape and comb, assemble plates into the electrophoresis chamber, add 1× TBE buffer to the chamber, and prerun gel at 700 V for 30 min. Before loading, heat the DNA marker for 5 min and the PE samples for 1 min in a 100 °C heating block, briefly spin the samples, and load 5 μl of each sample into the wells which need to be cleaned just before loading, for example with a Pasteur pipette. Run the gel at 700 V until the fast blue reaches the bottom of the gel. Disassemble glass plates, pick up the gel on Whatman paper and dry it in a vacuum gel dryer. Expose the gel to an X-ray film in a film cassette, best with an intensifying screen at −80 °C. Expect exposure times of 3–16 h. Develop film.

### 3.3 Promoter Pull-Down Assay

When designing primers to amplify a promoter region, it is important to provide ~20 bp of additional sequence around the known promoter elements to provide a DNA platform for factor binding. Moreover, if the sequence elements of a promoter have been characterized, a most valuable control for this assay is to analyze a comparable DNA in which the promoter element(s) have been mutated. Once the assay is established for a particular transcription factor, a refined mutational analysis of the promoter region can reveal the critical nucleotides for factor binding.

1. Generate biotinylated promoter DNA by PCR in which one of the oligonucleotides carries a biotin group at its 5′ end. Gel-purify the amplification product, using the QIAquick gel extraction kit.
2. For each assay, couple 500 ng of the PCR product to 100 μg of Streptavidin Dynabeads M-280 as follows: Vortex manufacturer’s beads vial for 30 s and transfer 10 μl of beads solution (10 μg/μl) to a new microcentrifuge tube. Wash the beads with 0.8 ml of 1× B&W buffer, collecting the beads on a magnet for 2 min. Resuspend beads in 100 μl of 2× B&W buffer and add DNA resuspended in 100 μl of water. Slowly rotate the tube for 15 min at room temperature. Collect the biotinylated DNA-coated beads and wash three times with 0.8 ml of 1× B&W buffer.
3. Block the beads with 0.5 ml of TK_20_ buffer, containing 5 mg/ml bovine serum albumin and 5 mg/ml PVP10, by slowly rotating the tube for 30 min at room temperature. Wash the beads twice with 0.5 ml TK_20_ buffer.
4. Set up an in vitro transcription reaction as described in Subheading 3.2.1, **steps 2** and **3** using the DNA-coated beads as template (*see* Note 11). Incubate the reaction for 15 min on ice and for 15 min at 27 °C. Wash the beads three times with 0.5 ml of TK_20_ buffer and once with 0.5 ml of TN_40_ buffer (*see* Note 12).
5. Elute proteins in 40 μl of 1× SDS loading buffer at 70 °C for 5 min and analyze sample by standard immunoblotting.

## Footnotes

1. We standardly use the 15-ml Dounce homogenizer because it is less likely to break. However, the extracts were originally established with the smaller 7-ml “tight” Dounce homogenizer from Wheaton and this homogenizer is more suitable when the PCV of pelleted cells is in the range of 2–4 ml.

2. In other systems, the “viscous” ingredient of transcription buffers most often is glycerol and not sucrose. However, trypanosomatid extracts typically harbor glycerol kinase activity which, in the presence of even trace amounts of glycerol, can rapidly deplete ATP pools [24].

3. We have always used circular plasmid DNA in transcription reactions because linearization of a plasmid template just downstream of the tag did not result in a transcription signal, that is, primer extension product. However, we learned that trypanosome RNA pols fall off linear templates before reaching the linearization site. Accordingly, transcription signals were achieved when the plasmid was linearized ~40 bp downstream of the tag (A.G., unpublished results). It is therefore likely that PCR-amplified DNA templates will work in these systems.

4. Four liters of procyclic trypanosome culture will produce the maximal amount of broken cell suspension from a 5 L glass flask and allow for the use of the sturdy 15 ml-glass Dounce homogenizer. However, 2–3 L (2–3 × 10^10^ cells) of culture is sufficient when the 7-ml Dounce homogenizer is used (*see* Note 1).

5. While the 40-μl reaction described has resulted in robust transcription signals, downscaling the reaction to 25 μl has worked, too [29]. It is recommended when extract is precious. For example, it requires 2 L of *T. brucei* bloodstream form culture to prepare extract on the small scale.

6. The reactions work best if they contain a total of 40 μg/ml plasmid DNA. However, individual templates have different concentration optimums in different extracts. In most cases, the optimum is 7.5 μg/ml; therefore, nontemplate plasmid DNA such as pUC19 needs to be added for optimal activity. The extra DNA likely sequesters nonspecific DNA-binding activities that can interfere with transcription. With the exception of the GPEET-trm template which works best at 40 μg/ml in extracts prepared from procyclics, template concentrations above 25 μg/ml were found to be deleterious.

7. Transcription reconstitution is most convincing when the factor for add back is expressed and purified from a different system (e.g., *Escherichia coli* or wheat germ extract). However, this is only feasible if the factor comprises not more than one or two proteins. In cases of larger protein complexes such as TFIIH (nine subunits) or CITFA (eight subunits) we have used endogenous factor of high purity that was isolated from extract by tandem affinity purification. Although the protein amounts necessary for successful transcription reconstitution may vary among factors, amounts in the range of 10–100 ng were sufficient in our hands.

8. Inactivation of a factor by an antibody is not a given because the antibody needs to bind to and block a functionally important domain. Since a polyclonal antibody/immune serum likely recognizes more than one epitope on a factor, its blocking potential is greater than that of a monoclonal antibody.

9. The generation and detection of radiolabeled primer extension products as described here is very sensitive and requires relatively few procedural steps. On the other hand, radiolabeled nucleotides are expensive and short-lived, and working with radioactive materials is tightly regulated. Thus, nonradioactive labeling methods such as the use of biotin- or fluorescent-labeled primers may be suitable alternatives [35–37].

10. If primer extension products smaller than 70 nt are investigated, an 8% PAA/50% urea gel will offer better resolution.

11. The promoter pull-down assay will most likely be successful under conditions of a functional transcription reaction which ensure that relevant transcription factors interact with the promoter. However, a transcriptionally active extract is not a prerequisite for this assay and promoter–factor interaction may also occur in inactive extracts.

12. The specified buffers provide the lowest stringency conditions we used successfully in this assay. However, once a pull-down has been achieved, increasing the salt concentration of the TK and TN buffers and/or adding a non-ionic detergent will increase the specificity of the assay. For example, CITFA was pulled down using 0.1% Tween 20-containing TK_40_ and TN_80_ buffers, in which the KCl and NaCl concentrations of the specified TK_20_ and TN_40_ buffers were increased to 40 and 80 mM, respectively, whereas a TK_150_ buffer abolished CITFA binding to its cognate promoter.
